# An Evidence-Based Cognitive Stimulation and Physical Activity Intervention to Delay Aging-Related Decline: Protocol for a Randomized Controlled Trial

**DOI:** 10.2196/88268

**Published:** 2026-07-08

**Authors:** Yook Chin Chia, Eden Ngah Den Low, Michael Jenkins, Yun Li Lee, Tin Tin Su, Rozainee Khairudin, Khazriyati Salehuddin, Santha Vaithilingam, Jactty Chew, Wei Ling Lim, Min Hooi Yong, Alexandre Schaefer

**Affiliations:** 1 Department of Clinical Medicine and Surgery, Jeffrey Cheah Sunway Medical School, Faculty of Medical and Life Sciences Sunway University Bandar Sunway, Selangor Malaysia; 2 Department of Primary Care Medicine, Faculty of Medicine University of Malaya Kuala Lumpur, Kuala Lumpur Malaysia; 3 Ageing, Health and Well-Being Research Centre, Jeffrey Cheah Sunway Medical School, Faculty of Medical and Life Sciences Sunway University Bandar Sunway, Selangor Malaysia; 4 Department of Biomedical Sciences, Jeffrey Cheah Sunway Medical School, Faculty of Medical and Life Sciences Sunway University Bandar Sunway, Selangor Malaysia; 5 Department of Psychology, Faculty of Science and Engineering University of Nottingham Malaysia Campus Semenyih, Selangor Malaysia; 6 Research Centre for Human-Machine Collaboration (HUMAC), Faculty of Engineering and Technology Sunway University Bandar Sunway, Selangor Malaysia; 7 Global Population Health, Jeffrey Cheah School of Medicine and Health Sciences Monash University Malaysia Bandar Sunway, Selangor Malaysia; 8 Department of Psychology, Faculty of Social Sciences and Leisure Management Taylor's University Subang Jaya, Selangor Malaysia; 9 Centre for Research in Language and Linguistics, Faculty of Social Sciences and Humanities National University of Malaysia Bangi, Selangor Malaysia; 10 Sunway Institute for Global Strategy and Competitiveness Sunway University Bandar Sunway, Selangor Malaysia; 11 Department of Psychology, School of Law and Social Sciences, Faculty of Management, Sciences and Engineering University of Bradford Bradford United Kingdom; 12 Department of Psychology, Faculty of Medical and Life Sciences Sunway University Bandar Sunway, Selangor Malaysia

**Keywords:** cognitive decline, physical decline, intervention-control study, biological and neurocognitive markers, aging, healthy aging, cognitive aging

## Abstract

**Background:**

The aging population presents both opportunities and challenges. Both global and Malaysian statistics have shown that an increase in longevity is also marked by an increase in the time spent in poor health. A key measure of healthy aging is the ability to lead an independent life. This has implications not only for the individual’s quality of life but also for society as a whole; loss of independence with age is associated with increased economic burden and reduced workforce productivity. Understanding and subsequently addressing these age-related declines (slowing or reversing them) is critical in improving the health and societal challenges faced by older adults. However, most studies are focused on Western populations. The scarcity of interventions tailored to multiethnic Asian populations is compounded by the fact that existing measurements rely heavily on Western-designed psychometric instruments, which frequently fail in capturing true cognitive health because of large cultural and educational gaps.

**Objective:**

We aim to examine whether our long-term intervention packages can, over 4 years, significantly slow down the normal rate of aging-related decline in cognitive function and brain structure and function, as well as assess changes in aging-related salivary biomarkers. We will also measure the economic impact of such interventions in a cost-benefit analysis.

**Methods:**

We propose 3 ecologically valid intervention packages (cognitive stimulation, physical activity, or both combined) and aim to assess them against a control group. We will target a sample of 400 participants as representative of the population of community-dwelling aging citizens in Malaysia (aged 60 years and older). The 5 projects of this study examined (1) psychology (social interaction and emotional well-being); (2) neuroscience, looking at neural markers of cognition (magnetic resonance imaging and electroencephalogram); (3) decision-making (risk and challenges in decision making); (4) economics (cost-benefits and effectiveness of the study interventions); and (5) biological markers of aging, using salivary samples. The Sunway University Institutional Review Board has reviewed and approved the study (SUREC2020/039).

**Results:**

Briefly, primary outcomes will include changes in cognitive scores (Montreal Cognitive Assessment), changes in cognitive behavioral measurements, changes in electroencephalogram and structural magnetic resonance imaging, changes in everyday problem-solving and Iowa gambling task scores, changes in salivary biomarkers (lactoferrin, C-reactive protein, and telomere length), and cost-benefit analysis of the intervention. The study grant was awarded in August 2019, with recruitment starting in May 2022 and concluding in July 2023. In 2023, the intervention phase began and is currently ongoing, with the first publications of outcomes expected in 2026.

**Conclusions:**

The effectiveness of these interventions will be examined from the perspective of multiple disciplines, including psychology, neuroscience, biology, and economics. We anticipate that the results of our study will be of interest to both the academic and general community and will hopefully influence policymaking. We hope that this study will provide robust and impactful, evidence-based insights on healthy aging and, thus, contribute to improving the overall quality of life associated with aging.

**Trial Registration:**

ClinicalTrials.gov NCT06376656; https://clinicaltrials.gov/study/NCT06376656

**International Registered Report Identifier (IRRID):**

DERR1-10.2196/88268

## Introduction

### Background

There is a consensus from both cross-sectional and longitudinal research on the pattern of changes associated with aging, especially among older adults. With aging comes a natural decline in muscle mass and strength, leading to risks such as falls, fractures, disability, and a loss of independence [1]. Along with physical decline, research evidence has shown nearly a linear decline in cognitive function based on measures representing efficiency or effectiveness of information processing [2]. Research conducted in the United Kingdom and Malaysia has shown much poorer performance in theory of mind and working memory in the Malaysian older adult population compared to the other 3 groups (British young and older adults and Malaysian young adults). Many of these findings are moderated by educational levels, which suggests that interventions aimed at older adults need to be targeted accordingly [3]. According to Carstensen [4,5], the sense of future time, particularly the approach of endings such as the end of life, elicits cognitive, emotional, and motivational changes [4,5]; for example, aging is associated with a trend toward favoring positive over negative stimuli in cognitive processing [6]. This potentially introduces risks regarding how older adults make important decisions that have financial consequences in their daily lives; this is supported by evidence from research linking age-related changes in brain activity and physiology with financial decision-making [7,8].

At present, there is promising evidence that cognitive training and physical activity may delay the decline of and possibly increase gray matter volume in the brain [9,10]. There is evidence suggesting that physical activity plays a critical role in preserving muscle mass, maintaining mobility [11], and enhancing neuroplasticity [12], whereas cognitive stimulation through activities that challenge attention, memory, and language supports mental agility and resilience [13]. Importantly, both physical and cognitive domains are closely linked to the performance of basic and instrumental activities of daily living, underscoring the need for interventions that integrate these components. However, these studies are often conducted in laboratories using traditional cognitive and memory tasks with little ecological validity and minimal consideration of general everyday life. Furthermore, most studies are conducted in the Western world; it is unclear whether such interventions are suitable for non-Western populations such as Asian individuals. In addition, most training interventions are conducted in brief timescales, and it is not clear whether these effects remain stable in the long term [14]. On the basis of systematic reviews by Masini et al [15] and Bibi et al [16], most interventions in older adults, including physical activity, cognitive training, or behavioral interventions, are conducted across durations ranging from 12 weeks to 12 months, with outcome assessments limited to immediately after the intervention or short follow-up periods (within 6 months). While there is a lack of universal agreement on the duration of long-term interventions, the long-term effects of physical activity are defined as at least 24 months of intervention and follow-up to better determine the effectiveness of measured physical activity [17]. Studies examining interventions using physical activity and cognitive training to promote healthy aging in older adult populations have mostly been conducted among Western populations [18-20], implying a potential research gap in studying the effectiveness of these interventions in older adults from non-Western populations (eg, Asian individuals) and exploring the cultural and socioeconomic determinants that may influence these populations’ participation and engagement [16,21,22]. Our own systematic review showed that physical activity interventions work best in combination with simultaneous cognitive stimulation interventions in terms of improving cognitive outcomes in older adults and that a longer duration matters (more than 20 sessions) [23]. Moreover, conducting interventions in communities improves ecological validity, particularly by placing age-related decline within the relevant sociocultural context, and has potentially a large social and economic impact in those communities. However, a major challenge for long-term interventions is engagement and acceptability, which depend largely on motivational factors. To increase participants’ engagement, socioculturally relevant motivational needs need to be considered, and cost-benefit analyses need to be included to understand the mechanisms of long-term voluntary engagement in such interventions.

In addition, the use of salivary biomarkers for aging is of interest due to their noninvasive nature and cost-effectiveness, along with the advancement of various analytical assays. Current research efforts focused on the identification of salivary biomarkers in the diagnosis and monitoring of disease progression may be useful to predict cognitive decline. Crucially, using these biomarkers to monitor intervention efficacy addresses a significant gap in the literature, as they are rarely applied as dynamic measures of progress. These biomarkers include lactoferrin, a potential predictor of early development of dementia and Alzheimer disease [24]; C-reactive protein (CRP), as elevations in its systemic concentrations are also associated with the risk of developing metabolic-related and neurodegenerative diseases [25,26]; and telomere length, a suggested candidate biomarker to indicate individual variability in aging and risk of specific health conditions in older adults [27,28].

**Figure 1:**
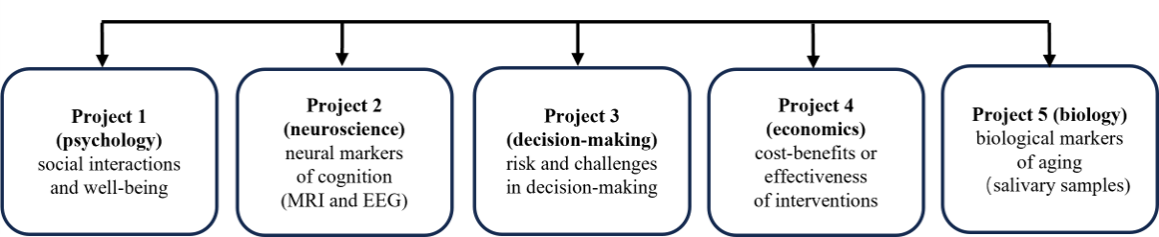
Overview of the study. EEG: electroencephalogram; MRI: magnetic resonance imaging.

The overarching aims of this project are to assess cognitive function across a broad range of measures, contextualize these measures within additional demographic and lifestyle data, implement and validate evidence-based interventions derived from theories of cognitive decline that incorporate both physical and cognitive activities, and assess the effectiveness of these interventions in modulating the trajectory of age-related cognitive decline in a socioeconomically and ethnically diverse Asian sample. The main objectives and specific objectives are summarized in the following sections. In this paper, we propose the comparison of 3 long-term interventions (cognitive stimulation, physical activity, and combined cognitive stimulation and physical activity) against a passive control condition. These interventions will be administered over an extended period (4 years). We hypothesize that these interventions can, over 4 years, significantly slow down the normal rate of aging-related decline in cognitive function and brain structure and function, and change the expression of aging-related salivary biomarkers. We predict that cognitive stimulation and physical activity will lead to a significant reduction in aging-related decline as compared to the nonintervention control. In addition, we predict that the combined cognitive stimulation and physical activity intervention will yield the most significant reduction in aging-related decline as compared to cognitive stimulation alone, physical activity alone, and the control condition. The overall structure of the program is shown in Figure 1. These 5 separate projects are interrelated not only because they involve the same participants but, more importantly, because they all aim to delay aging-related decline. Age-related decline is manifested in individuals’ cognitive and behavioral functions, neural markers of cognition, decision-making, and biological markers. The effectiveness of the cognitive and/or physical interventions will be measured (projects 1, 2, 3, and 5), and the economic impact of such long-term interventions in a cost-benefit analysis will also be evaluated (project 4).

### Main Objectives

The main objectives of this study are to (1) examine whether these interventions can, over 4 years, significantly slow down the normal rate of aging-related decline in cognitive function, physical performance, and brain activity and measure the economic impact of such interventions; (2) test hypotheses derived from aging-related theories; (3) provide robust, measurable evidence in both the short and long term and validate meaningful interventions (positive results or improvements in cognition, mental, and physical health that we are going to implement as being acceptable and sustainable in the long term); and (4) provide a quantifiable cost-benefit ratio for suggested solutions.

### Specific Objectives

There are 5 projects in this study. Briefly, we will examine older adults’ self-report and behavioral (task-based) markers of cognitive function (project 1), structural and functional brain measures (project 2), decisions impacting important and less important aspects of daily life (project 3), and cost-benefit analyses and economic gains and losses of the interventions (project 4), as well as identifying the effects of the interventions on aging-related salivary biomarkers (project 5). The specific objectives by project are summarized in Multimedia Appendix 1.

## Methods

### Trial Design

This quasi-randomized controlled trial has a longitudinal mixed-subject design for a total of 4 years (between-subject: intervention type; within-subject: repeated measures on various tasks and assessments). Participants will be quasi-randomly allocated to 1 of the 4 groups (cognitive stimulation, physical activity, combined cognitive stimulation and physical activity, and control) in a sequential cycle by experimenters to ensure balance in group sizes. Minor adjustments may be made for a small proportion of participants (anticipated to be fewer than 15% of the intervention sample) to accommodate logistical feasibility, such as availability during intervention sessions or access to required resources (eg, internet-enabled devices for online cognitive sessions). These adjustments will be applied only when necessary. Participants and experimenters will not be blinded to group assignment. Participants will be tested at baseline and every 6 months until the end of 4 years. Structural magnetic resonance imaging (MRI) will only be performed at baseline and at the end of the intervention period.

### Study Setting

A total of 400 Malaysian older adults aged 60 years and above will be recruited from the Klang Valley area. All physical activity interventions (with the exception of the forest walk and city walk) will be carried out in person within the compounds of Sunway University, whereas the cognitive stimulation interventions will be conducted online.

### Inclusion Criteria

The inclusion criteria are (1) healthy status (including those seeking regular medical attention); (2) some form of mobility (ability to walk short distances for at least 3 m); and (3) ability to communicate in at least one of the following languages: English, Malay, Mandarin, or Tamil.

### Exclusion Criteria

The exclusion criteria are (1) history of stroke, (2) diagnosis of a neurodegenerative disease (such as Alzheimer disease or Parkinson disease), (3) diagnosis of psychiatric disorders and/or being currently on psychiatric medications, (4) uncorrected auditory and/or visual impairments, (5) immobility or requirement for full-time caregiver assistance, (6) comprehension impediments or a Montreal Cognitive Assessment (MoCA) [29] score of less than 13.

Although the suggested cutoff score for the MoCA is 26, this might not be a true reflection of mild cognitive impairment in every country; hence, modifications to the cutoff score can be made according to the cultural and educational backgrounds of individuals in a particular country [30]. A systematic review of studies that used the MoCA in Southeast Asia reported that, in countries such as Malaysia, Singapore, China, and Taiwan, researchers used a cutoff ranging from 13 to 26 to take the educational background in these regions into account [31].

### Recruitment

Recruitment of participants will be conducted by advertising on social media, as well as visiting community clubs and meeting with community leaders in different housing areas across different socioeconomic strata. We refer to this cohort as the MyAgeWell cohort.

### Ethical Considerations

Ethics approval has been obtained from the Sunway University Institutional Review Board (reference SUREC2020/039; approval date: April 3, 2020). No substantive ethical issues have been identified with the conduct of our study. The protocol has also been registered on ClinicalTrials.gov with registration number NCT06376656 [32]. Participants will receive compensation for their participation in baseline assessments, interim measurements, and intervention sessions.

Participants will be recruited after providing written informed consent. Participants will retain the right to voluntarily withdraw from the study at any time. Unique codes will be assigned to each participant to ensure confidentiality. Records will only be accessible to involved researchers. Research findings from this study will be disseminated through presentations at conferences and publications in peer-reviewed academic journals.

### Baseline Measurements

The MoCA scores obtained by participants during their recruitment will serve as their baseline cognitive scores. In addition, cognitive behavioral measurements (2-back memory task [33], Wisconsin card sorting task [34,35], Tower of London task [36], and go/no-go task [37]), an electroencephalogram (EEG; resting state and during the go/no-go task), structural MRI, the everyday problem-solving task [38], and the Iowa gambling task [39] will be administered at baseline.

Participants will also answer a baseline questionnaire with regard to their demographics (gender, age, ethnicity, religion, spoken languages, marital status, occupation, educational level, savings, debts, assets, and social activities), health conditions (extracted questions from the Mini Nutritional Assessment [40] and Pittsburgh Sleep Quality Index [41] and voluntary disclosure of medical conditions), physical activity status (International Physical Activity Questionnaire) [42], mental health status (selected questions from the Center for Epidemiologic Studies Depression Scale [43] and State-Trait Anxiety Inventory [44]), Satisfaction With Life Scale [45], and life-course socioeconomic status (childhood, adulthood, and late adulthood). Participants may choose not to answer specific questions. In addition, saliva samples will be collected.

### Intervention

The intervention phase is intended to take place over a 4-year span, with interim measurements collected every 6 months. To maintain participant engagement, the intervention phase will be split into sequential activities, each carried out for a duration of 6 months.

#### Intervention Group 1: Cognitive Stimulation

The cognitive stimulation intervention will comprise psychoeducation, video gaming, jigsaw puzzle solving, and new language learning. These activities target multiple domains of cognition (eg, memory, attention, and executive function) and are both stimulating and practical to maximize ecological validity and adherence. For 6 months, participants will attend a weekly hour-long session of one of the aforementioned activities. Every 6 months, the participants in the cognitive stimulation group will alternate among the 4 above-mentioned cognitive stimulation activities until the end of the 4-year intervention period.

#### Intervention Group 2: Physical Activity

The physical activity intervention will comprise walking, dancing (Zumba), resistance training, and qigong sessions. These physical activity interventions are age appropriate and ecologically sound, with the potential to be adopted as part of participants’ healthy aging practices. For 6 months, participants will attend a weekly hour-long session of one of the aforementioned activities. Every 6 months, the participants in the physical activity group will alternate among the 4 above-mentioned physical activities until the end of the 4-year intervention period.

#### Intervention Group 3: Combined Cognitive Stimulation and Physical Activity

Participants in the combined cognitive stimulation and physical activity group will attend 2 sessions of interventions per week (a session of cognitive stimulation and a session of physical activity).

#### Control Group

Participants in the passive control group will not attend any intervention sessions but will attend the interim sessions every 6 months.

#### Record of Attendance

Attendance to each intervention session will be recorded, and participants will be asked to submit a weekly Google Form to record details of activities they have done over the previous week in their free time, which include physical, cognitive, social, and leisure activities not part of the intervention. This will be helpful for us to observe and control the frequency of other nonintervention activities while participants take part in the intervention.

#### Interim Measurements

Throughout the intervention period, participation in intervention components will be assessed via both self-reports and interviewer-administered surveys. The MoCA, cognitive behavioral measurements, EEG, and saliva collection will be repeated every 6 months.

#### End of Intervention Period

**Figure 2:**
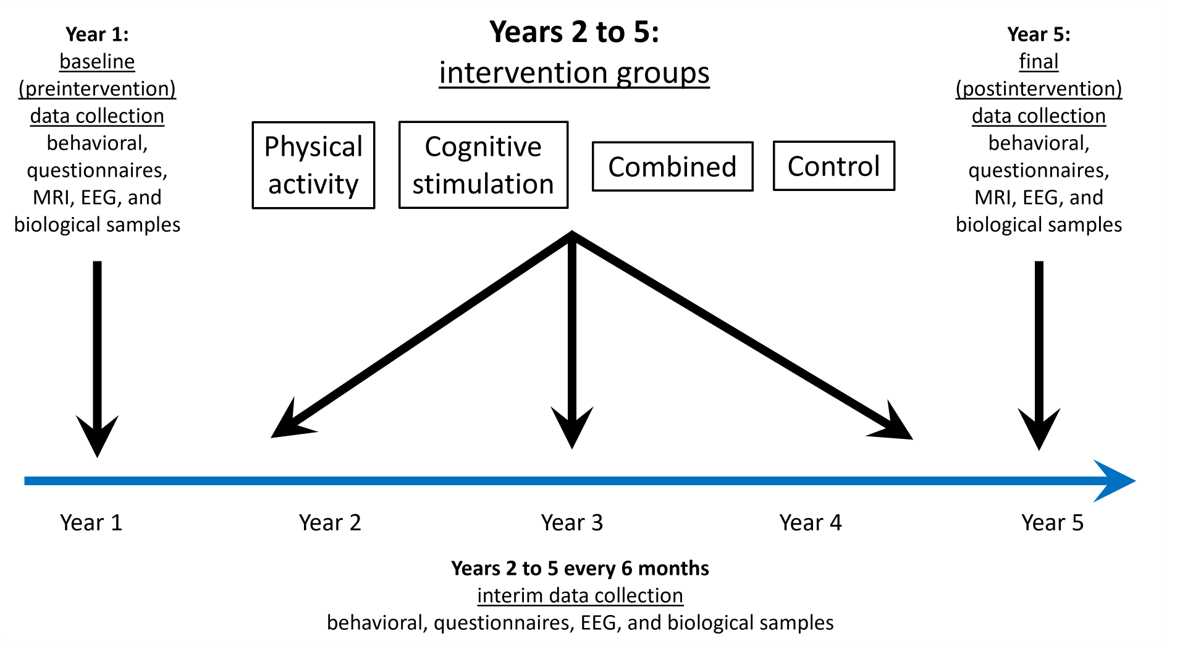
Interventions and data collection. EEG: electroencephalogram; MRI: magnetic resonance imaging.

The MoCA, cognitive behavioral measurements, EEG, and saliva collection will be repeated at the end of the intervention period. MRI will only be performed during the last interim period (after 4 years) for all participants. A summary is provided in Figure 2.

### Sample Size

Simulation-based power analysis was performed using the *superpower* R package [46] in RStudio (version 2023.06.1; Posit PBC). This analysis determined that a sample size of 80 participants per intervention group would be sufficient to detect a medium effect size (η^2^_p_=0.06) in a 2 × 2 mixed-design interaction with 86.6% power. The 2 × 2 design was chosen as a comparison of pre- and postintervention measures between an intervention and control group, providing a basic assessment of whether a specific intervention is effective. This assumes a correlation of at least 0.8 between repeated measures. We calculated that a sample of 400 would be necessary to reach our goals, given that we expect an attrition rate of 20% (80/400 participants) between the pre- and postintervention phases for several reasons (ie, death, decline in health, and dropouts in training sessions).

### Data Collection

#### Overview

A copy of a personal information sheet and a consent form will be given to participants to inform them about the study and obtain consent. Participants will answer a baseline questionnaire using a Google Form. A researcher will help administer the MoCA to the participants based on their preference (online or in-person session). Structural MRI will be used to determine brain structure (gray matter volume), and EEG will be used to determine brain function (electrophysiological activity), both while participants are at rest and while they complete a cognitive task (the go/no-go task). After a brief rest, participants will be asked to rinse their mouths with water to proceed with saliva collection. They will then be seated comfortably and asked to lean forward and not swallow. After 5 minutes, saliva will be collected in a collection tube via passive drooling [47].

#### Structural MRI

Structural MRI data will be acquired using a 3.0-Tesla scanner. High-resolution T1-weighted images will be obtained for gray matter assessment, with isotropic voxel resolution suitable for voxel-based morphometry (eg, 1 × 1 × 1 mm^3^). Preprocessing will include quality inspection, tissue segmentation, spatial normalization, and smoothing. Gray matter volume will be quantified via voxel-based morphometry, with longitudinal analyses conducted after the intervention using general linear models adjusting for covariates (age, sex, and intracranial volume). These measures are interpreted as indexes of cumulative, longer-term neural adaptation.

#### EEG Measurement

EEG data are acquired using a portable multichannel EEG system with dry electrodes arranged according to the International 10-20 system, allowing for coverage of the frontal, central, parietal, temporal, and occipital regions. This configuration is suitable for assessing large-scale cortical activity associated with executive control, attention, and response inhibition. EEG signals are sampled at a temporal resolution of 500 Hz, allowing for reliable characterization of both oscillatory activity and task-related event-related potentials (ERPs) in accordance with manufacturer specifications. Resting-state EEG is recorded for a total of 4 minutes under counterbalanced eyes-open and eyes-closed conditions segmented into 1-minute blocks to minimize fatigue and habituation effects. Participants are instructed to remain relaxed and awake and avoid goal-directed cognitive activity during recording.

EEG preprocessing includes band-pass filtering using standard frequency ranges for resting-state and task-based analyses, as well as artifact handling procedures comprising visual inspection and removal of segments contaminated due to eye blinks, muscle activity, movement, or electrode instability. Sessions with excessive artifacts are documented and excluded as appropriate to ensure data quality. During the go/no-go task, EEG is recorded concurrently with behavioral performance. Task-related analyses focus on ERPs associated with response inhibition and cognitive control, specifically, components time-locked to stimulus presentation during go and no-go trials. ERP waveforms are derived by averaging across trials after artifact rejection, with component amplitudes and latencies extracted from fronto-central electrode sites commonly implicated in inhibitory control processes. Longitudinal EEG changes are examined at 6-month intervals using mixed-effects modeling, which accommodates repeated measurements, missing data, and interindividual variability. This analytic approach enables the examination of within-subject changes over time while accounting for relevant covariates, thereby providing sensitive indexes of functional neural adaptation associated with the interventions.

### Data Management

Raw data (including any video recordings) will be kept confidential. Only those directly involved in the project (ie, the researchers and data coders) will have access to the data, which will be stored on password-protected computers and data storage facilities. The results of the project may be published and will be available in the Tun Hussein Onn Sunway Library (Malaysia), but every attempt will be made to preserve participants’ anonymity.

### Cost-Benefit Analysis

We will adopt the integrated cost-benefit analysis and social return on investment approach to comprehensively capture the multidimensional benefits of the cognitive stimulation, physical activity, and combined interventions among older adults in Malaysia. This approach also accounts for broader societal gains for caregivers, families, and the community. Costs will be classified into 5 domains, namely, staff training and other labor, intervention supplies and medical services, venue hire for intervention activities, transportation, and participant time, consistent with the literature [48].

### Statistical Methods

The primary analysis will be conducted using a repeated-measure linear mixed-effects model with time (baseline vs postintervention time point) as a within-subject factor and intervention type (cognitive stimulation vs physical activity vs combined vs control) as a between-subject factor. Participants will be included as a random effect to account for repeated measurements. Analyses will follow the intention-to-treat (ITT) principle, with baseline covariates such as age and sex included as fixed effects where appropriate.

Missing data will be handled using both ITT and per-protocol approaches. For participants with minimal missing data (<15%), analyses will primarily follow ITT principles without imputation, as this level of missingness is unlikely to bias the results. If missing data exceed this threshold, appropriate imputation methods, such as multiple imputation, will be applied. Sensitivity analyses will also be conducted to ensure robustness across both ITT and per-protocol analyses.

For exploratory purposes, paired 2-tailed *t* tests may be conducted to compare outcome values (before and after the intervention for each intervention phase, eg, telomere length), and one-way ANOVA may be used for simple between-group comparisons at a single time point. Results with a *P* value of less than .05 will be considered statistically significant, with appropriate attention to multiple testing for secondary analyses.

### Biological Samples

Saliva samples will be kept at −20 °C until further processing. Any remaining samples will be stored for further analysis. Changes in levels of human salivary CRP (mg/L) will be measured using salivary enzyme-linked immunosorbent assay kits. Changes in salivary telomere length (telomere-to–single-copy gene ratio) will be measured using quantitative polymerase chain reaction.

## Results

From the perspective of psychology, primary outcomes will be the changes in cognitive scores (MoCA), cognitive behavioral measurements, everyday problem-solving, and Iowa gambling tasks. From the perspective of neuroscience, primary outcomes will be the changes in EEG and MRI. From the perspective of economics, the primary outcome will be the cost-benefit analysis of the intervention. From the perspective of biology, primary outcomes will be the changes in salivary biomarkers (lactoferrin, CRP, and telomere length).

The study grant was awarded in August 2019. Participant recruitment was delayed due to the COVID-19 pandemic, as the target population comprised healthy older adults who were particularly vulnerable during periods of movement restrictions and heightened infection risk. Recruitment commenced in May 2022 and was completed in July 2023. A total of 400 healthy older adults were successfully enrolled. The intervention phase began in 2023 and is currently ongoing, with participants undergoing intervention activities and follow-up assessments at 6-month intervals throughout the study period. At the time of manuscript submission, follow-up data collection and data analysis are ongoing. Baseline characteristics and cross-sectional findings from the cohort have been reported separately, while analyses of intervention-related outcomes are currently underway. The first intervention outcome publications are anticipated from 2026 onwards as follow-up assessments are completed, and additional longitudinal data are available for analysis.

## Discussion

### Expected Findings

According to a statement released by Malaysia’s Chief Statistician in July 2019 [49], it is predicted that at least 15% of the total Malaysian population will be classified as aging, which is defined as older adults aged 65 years and older, by 2030. With a projected longer life span, there are economic, social, and individual burdens placed on society and the nation. Dependence on adult children creates an economic burden and may result in stress within families to care for older adults. Hence, the need to remain independent in their golden years is of paramount importance for older adults and their families. However, a significant knowledge gap persists regarding aging within Asian communities. While Western studies have shown promising evidence for cognitive and physical interventions, it remains unclear whether these models are culturally suitable or sustainable for a multiethnic Malaysian population. Furthermore, the reliance on Western-centric psychometric tools often fails to capture the true cognitive status of Asian cohorts. By integrating objective neuroimaging and salivary biomarkers, this study seeks to provide a high-resolution “ground truth” for cognitive health that transcends cultural and educational biases.

Outcome sensitivity varies by modality. EEG assessments will be conducted every 6 months over the 4-year study period, enabling detection of short- to medium-term functional neural adaptations associated with ongoing intervention exposure. Prior studies in older adults indicate that functional neural changes can occur following relatively modest intervention doses, whereas structural brain changes typically require greater cumulative exposure. For example, Yang et al [50] reported significant resting-state EEG changes (reductions in theta power and theta-to-beta ratio) after an 8-week intervention delivered 3 times per week, accompanied by improvements in global cognitive performance. Each session lasted approximately 60 minutes and included structured cognitive and physical activities. Similarly, Kang et al [51] observed significant functional MRI connectivity changes following a 4-week virtual reality–assisted cognitive training program delivered twice weekly for 20 to 30 minutes per session.

In contrast, structural MRI assessments capture longer-term neuroplastic changes and will be conducted after the full 4-year intervention period. Previous studies reporting structural MRI changes generally involve longer intervention durations and higher cumulative session numbers. For instance, Anderson-Hanley et al [52] and Suo et al [53] observed increases in regional brain volume or cortical thickness following interventions delivered 3 to 5 times per week for at least 3 months with continued maintenance up to 6 months or delivered twice weekly for 26 weeks, respectively. These studies typically involved session durations ranging from 20 to 90 minutes, supporting the interpretation that structural MRI markers are less sensitive to short-term interventions and primarily reflect cumulative neuroplastic adaptation.

Overall, the selected intervention dose and staggered assessment schedule are expected to capture early functional neural changes (EEG), as well as longer-term structural adaptations (MRI). The intervention frequency (once weekly) and session duration (60 minutes) delivered over an extended period are consistent with previously reported intervention doses and are ecologically feasible for community-based implementation, supporting sustained participation and potential continuation in daily life. Importantly, there is evidence from prior studies suggesting that functional neural changes (EEG) can be detected after intervention periods of 6 months or less, whereas structural brain changes (MRI) typically require substantially higher cumulative exposure. In this study, the 4-year intervention period corresponds to approximately 192 sessions, exceeding the cumulative dose reported in previous studies that observed structural MRI changes after approximately 72 sessions or more.

We acknowledge that participants in the combined cognitive stimulation and physical activity intervention group will receive greater weekly contact time than those in single-modality groups, which introduces a potential confounding effect. As such, group differences must be interpreted with caution as they may reflect either synergistic benefits of combining modalities or simply the increased intervention dose. Our design choice was intended to reflect real-world multimodal programs, which often require longer engagement, but we recognize that this limits causal inference. Future studies should equate contact time across groups or use factorial designs to disentangle modality-specific effects from dose-related influences.

The intervention frequency of weekly 1-hour sessions is designed as a feasible starter dose and behavioral catalyst for sedentary Malaysian older adults. Mechanistically, low-frequency regular stimulation can sufficiently modulate the systemic inflammatory environment. We anticipate observable changes in salivary biomarkers; specifically, salivary lactoferrin has emerged as a robust, noninvasive indicator of early neurodegeneration reflecting central neuroinflammatory status [24]. Furthermore, regular physical activity has been shown to significantly reduce CRP levels in older populations [54].

This study will provide evidence-based insights into the impact of cognitive and physical interventions on healthy aging within a multiethnic Asian population. Importantly, by moving beyond the use of culturally biased psychometric tools, the use of neuroimaging and salivary biomarkers can reveal how targeted interventions preserve brain structure and modulate systemic biomarkers.

### Strengths and Limitations

The longitudinal nature of our study will help us understand the effects of long-term cognitive stimulation and physical activity in slowing down the normal rate of aging-related decline. In addition, the study population comprises aging Malaysian citizens of varying ethnicities and socioeconomic statuses. This will provide a much needed Southeast Asian perspective on the concept of healthy aging. In addition, long-term interventions in the form of cognitive stimulation and/or physical activity that aim to be accessible and feasible for individuals will help our participants build long-term habits that might facilitate healthy aging. The idea of engaging in long-term, sustainable activities should be promoted generally. Moreover, the wide range of data used to assess healthy aging provides a comprehensive profile of cognitive and brain health, allowing us to assess both the general and specific impact of the interventions.

Our study participants will only comprise community-dwelling older adults living in an urban setting. Thus, the generalizability of the results to other populations, such as those living in residential care homes or in rural areas, may be limited.

## Appendix

Multimedia Appendix 1 List of specific objectives by project.

Multimedia Appendix 2 CONSORT-EHEALTH checklist (V 1.6.1).

## Abbreviations

CRP: C-reactive protein
EEG: electroencephalogram
ERP: event-related potential
ITT: intention to treat
MoCA: Montreal Cognitive Assessment
MRI: magnetic resonance imaging

## Data Availability

The datasets generated or analyzed during this study are available from the corresponding author on reasonable request.
