# Supplementary material for: An Evidence-Based Cognitive Stimulation and Physical Activity Intervention to Delay Aging-Related Decline: Protocol for a Randomized Controlled Trial
Source: JMIR Res Protoc. 2026 Jul 8;15:e88268. doi: 10.2196/88268 (PMC13392532; doi:10.2196/88268)
Supplement: Multimedia Appendix 1 [file resprot_v15i1e88268_app1.pdf]

Multimedia Appendix 1

Table S1. List of specific objectives by respective projects.

Project 1: Social cognition and executive function aspects for social interaction and well-being	
1	To explore the effectiveness of intervention type on social cognition, executive function and well-being across time,
2	To explore other mediating/moderating social factors that influences the effectiveness of intervention type,
3	To examine if socioeconomic status modulates the effects of intervention (cognitive stimulation, physical activity) on changes in executive function, well-being, and social cognition,
4	To examine the relationships between social cognition, executive function, and well-being with neurological, and other biological markers.
Project 2: Modulating neural markers of age-related decline in cognitive function using magnetic resonance imaging (MRI) and electroencephalogram (EEG)	
1	To examine the effects of two types of intervention on grey matter volume change over four years, in an ageing population,
2	To examine the effects of two types of intervention on neuropsychological test performance change over four years, in an ageing population,
3	To examine the effects of two types of intervention on changes in brain event-related potentials reflecting selected cognitive processes relevant to ageing,
4	To examine if socioeconomic status modulates the effects of intervention on changes in neural and cognitive markers of ageing.
Project 3: Risks and challenges in making decisions	
1	To examine and explore the effectiveness of intervention type (physical activity, cognitive stimulation) on decision-making behaviors across time,
2	To examine the relationships between decision-making with other projects e.g. neurological and biological markers, social cognition,
3	To investigate whether socioeconomic status modulates the effects of intervention (physical activity, cognitive stimulation) on changes in decision making behaviors.
Project 4: Cost benefit analysis on interventions targeted for the elderly	
1	To determine the relative financial and economic efficiency economic benefits and costs of the intervention programs,
2	To establish a framework for determination of the explicit and implicit costs and benefits associated with the conduct of the intervention programs,
3	To suggest an appropriate mix of strategies and the proper solution for allocation of scarce resources,
4	To provide an overview of the total amount of resources needed for start and possibly expansion of the intervention projects.
Project 5: Salivary biomarkers in executive control and well-being in ageing population	
1	To investigate effects of different intervention types on levels of lactoferrin, C-reactive protein (CRP) and shortening of telomere length (TL) in older adults,
2	To develop the predictive value of salivary biomarkers in age-related decline,

3 To explore the relationship and predictive factors between salivary biomarkers and ageing-related decline identified in behavioral and neurological markers.
